# Recent development of mass spectrometry and proteomics applications in identification and typing of bacteria

**DOI:** 10.1002/prca.201500086

**Published:** 2016-02-02

**Authors:** Keding Cheng, Huixia Chui, Larissa Domish, Drexler Hernandez, Gehua Wang

**Affiliations:** ^1^ National Microbiology LaboratoryPublic Health Agency of CanadaWinnipegManitobaCanada; ^2^ Department of Human Anatomy and Cell Sciences, Faculty of MedicineUniversity of ManitobaWinnipegManitobaCanada; ^3^ Henan Centre of Disease Control and PreventionHenan ProvinceP. R. China

**Keywords:** Bacteria, Identification, Mass spectrometry, Typing

## Abstract

Identification and typing of bacteria occupy a large fraction of time and work in clinical microbiology laboratories. With the certification of some MS platforms in recent years, more applications and tests of MS‐based diagnosis methods for bacteria identification and typing have been created, not only on well‐accepted MALDI‐TOF‐MS‐based fingerprint matches, but also on solving the insufficiencies of MALDI‐TOF‐MS‐based platforms and advancing the technology to areas such as targeted MS identification and typing of bacteria, bacterial toxin identification, antibiotics susceptibility/resistance tests, and MS‐based diagnostic method development on unique bacteria such as *Clostridium* and *Mycobacteria*. This review summarizes the recent development in MS platforms and applications in bacteria identification and typing of common pathogenic bacteria.

**Abbreviations**
AFB: acid‐fast bacillus
ESI‐HRMS: ESI high‐resolution MS
GBS: Guillain–Barré syndrome
MA: mycolic acid
MLST: multilocus sequence typing
MS‐H: MS‐based H typing
TB: tuberculosis
UPLC‐MS/MS: ultraperformance LC‐MS/MS

## 1 Introduction

**Figure 1:**
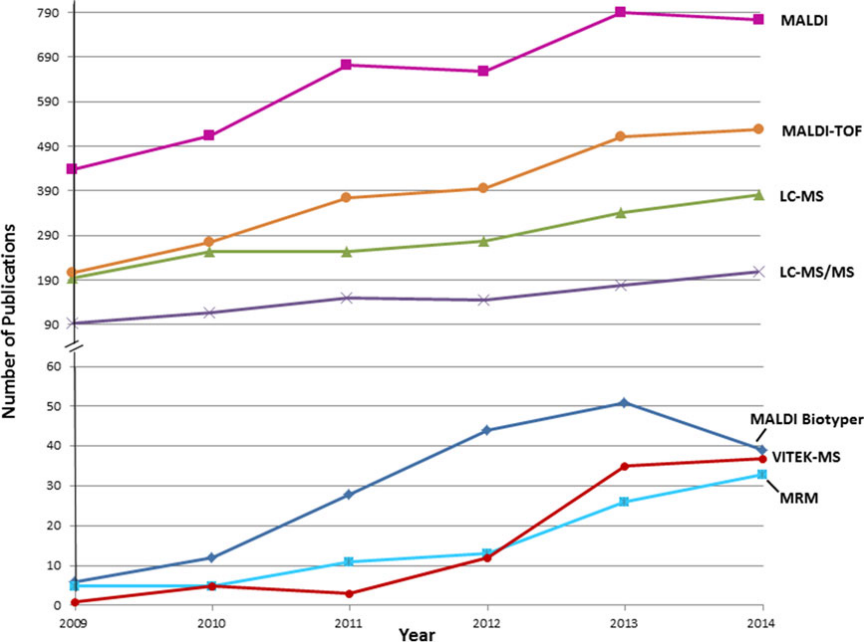
Literature report number with different key words searched in PubMed from year 2009–2014 for bacteria study.

MS and proteomics are gaining popularity in bacterial research and applications, especially after U.S. Food and Drug Administration approval of two MALDI‐TOF‐MS‐based platforms 1, 2. From the literatures available, it is obvious that this is an active area. We can see that from January 2009 to December 2014, scientists and medical professionals moved more toward MS approaches using platforms such as MALDI‐TOF‐MS or LC‐MS/MS, through mass fingerprinting or peptide sequencing (Fig. 1). There are also increasing occurrences where scientists have chosen MS methods and platforms tailored to fit the specific needs of their projects or molecules of interest, whether it is to target the slow‐growing *Mycobacteria* or to detect molecules such as antibiotics and related metabolites, by targeted LC‐MS/MS such as MRM or high‐resolution MS. With numerous types of MS instrumentation and several platforms available for use, together with the growing sensitivities and resolving powers of MS instruments on biomolecules, hundreds of new reports on bacterial identification and typing come out each year. Currently, MS has a wide range of applications from fast bacterial identification in food‐borne disease outbreak, water quality control, antibiotics susceptibility/resistance tests, rapid infectious disease diagnosis, to biomarker discovery that may help accurately identify closely related organisms. Some MS methods have become routine microbiological procedure in hospitals and research institutes in an effort to reduce turnaround times, costs, and overall labor 3. Even so, this is still a new area for many clinical microbiology labs, and there is still great potential for MS application in bacteriology research and diagnostic method development. As bacterial identification and typing occupy a major portion of time and work in clinical microbiology labs, this review will focus on that particular area of MS application. The review will first outline the commonly used MS platforms that are currently applied in the field and related methods in general, followed by highlighting some recent applications on noteworthy pathogenic bacteria with various MS platforms. Our first‐hand experience will also be integrated in the review. The MS platforms reviewed here include MALDI‐TOF‐MS‐based mass pattern and fingerprinting, LC‐MS/MS‐based peptide sequencing for bacteria identification at protein sequence level, and targeted LC‐MS/MS for molecular identification and quantitation of molecules of interest. The bacterial MS applications reviewed in this paper include those on common pathogenic bacteria such as *E. coli, Salmonella, Campylobacter, Clostridium, Listeria, Mycobacteria*, and a category of “other” bacteria. Finally, the overall impressions of MS usage in this area are discussed and future directions are predicted.

**Figure 2:**
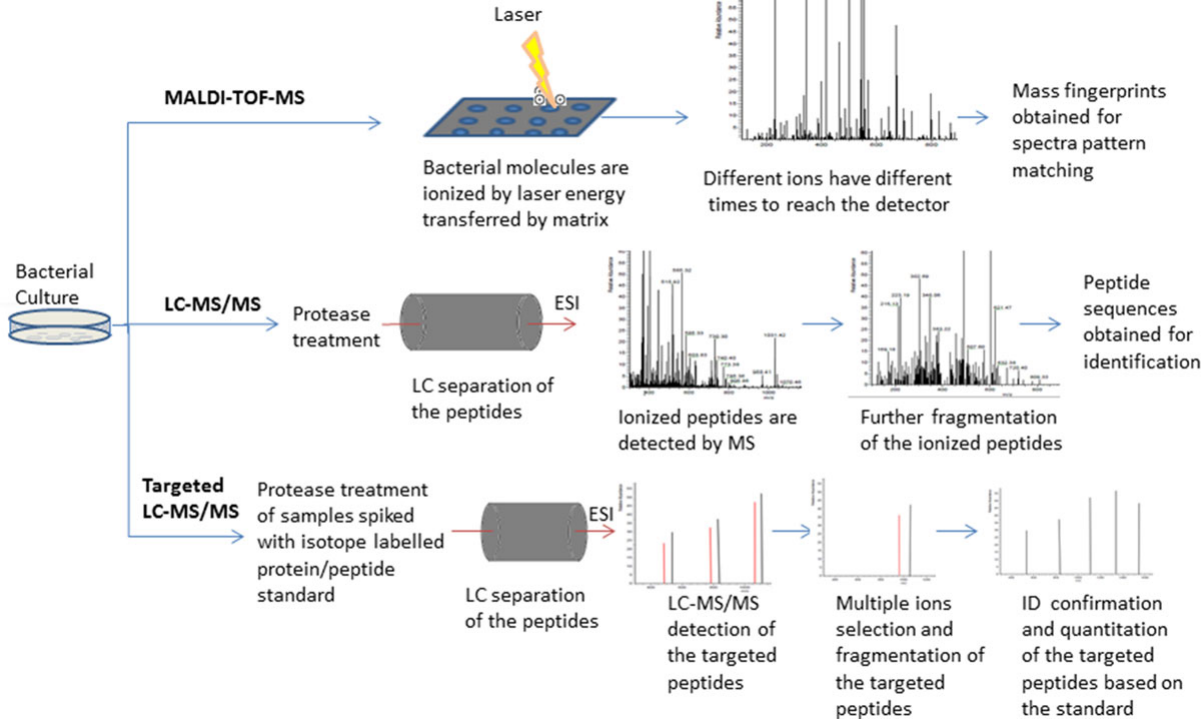
Common platforms of MS for bacterial identification and typing. ID, identification.

## 2 Common MS platforms in bacteria identification and typing (Fig. 2)

### 2.1 Mass pattern and spectra comparison by MALDI‐TOF‐MS

The U.S. Food and Drug Administration approved MALDI Biotyper and VITEK MS are both MALDI‐TOF‐MS‐based instruments and platforms by ionizing extracted molecules of whole cell culture without specific protease treatment. The bacteria culture is normally treated with strong solvent such as 1% TFA in 50% ACN 4 or 70% formic acid followed by 50% ACN 5 and spun down. Extracted molecules will be mixed with chemicals (matrices) such as CHCA and loaded onto MALDI plate for MS detection. The sample spots will be shot by laser energy and mass spectra, represented by *m/z*, will be obtained 4, 5, 6. Signals (spectra) from MS analysis will be compared against all of the different MS patterns/fingerprints that are harbored in the database (spectra library), i.e. mass fingerprints of well verified known bacteria stored in the database are compared with the spectra obtained from the sample. The identity of the microorganism will be based upon which set in the database provides the best match with the inputted spectra obtained from the sample. Bacteria culture can also be directly smeared onto the MALDI plate and then covered by the matrix of choice 6. Each matching spectra result is a potential identification and will be given a confidence score. Generally, a score equal or greater than 2 is considered a confident and correct identification 6. The scoring can be further categorized: anything below 1.7 being considered unreliable, a score from 1.7 to 1.9 indicating probable genus identification, 2.0 to 2.29 indicating a confident genus identification, and 2.3 to 3.0 indicating highly confident species identification 7. Some labs have also performed bacterial identification straight from positive blood culture by spinning down the blood cells first with low speed and then collecting the bacteria from the supernatant with higher speed. The bacterial pellet was then washed with water to reduce some interference from blood culture, and was finally treated with solvent such as 70% formic acid to extract some molecules out for MALDI‐TOF‐MS analysis 8, 9, 10. Although lower confidence scores may happen due to occasional polymicrobial samples 8 or lower bacteria number collected from the blood culture 9, 10, this is certainly faster than pure cultured‐based approach. Kroumova et al. has explored a rapid media cultured‐based enrichment approach after blood culture to decrease possible interference from blood culture and increase bacteria amount, and excellent MALDI‐TOF‐MS result was obtained 11.

Since Biotyper‐ or VITEK‐MS‐based spectra pattern comparison does not involve any protease treatment, the exact composition of the spectra is unknown. However, the spectra are often very unique for most bacteria, especially at genus level 12, 13, 14, 15. Statistical methods such as phyloproteomic principal component analysis can be used to find the patterns and unique peaks of individual strains 4, 6, and software such as Samaris can be used for strain identification by comparing the calibrated mass spectra to the reference spectra 12. Although pure cultures produce more stable and consistent result 14, 15, culture conditions may still affect the quality of the spectra and related identification 13. Interestingly, a recent report showed that MALDI‐TOF‐MS platform could also be used for antibiotics resistance test by growing bacteria in a media containing normal and isotope‐labeled amino acid lysine with and without antibiotics. The mass shift of many MS peaks, summarized through bioinformatics tools, indicated the capability of cell growth in the presence of antibiotics signifying antibiotic resistant 16.

### 2.2 Mass fingerprinting for molecules of interest by MALDI‐TOF‐MS or LC‐MS/MS

PMF is another form of mass fingerprinting on peptides, often obtained after protease treatment. The experimental spectra of masses are compared to the theoretical spectra of masses based on the protease digestion patterns, instead of a spectra library, and allow for the identification of a protein or molecules of interest 17. The proteins are normally prepared by an enrichment process such as gel electrophoresis or chromatography, after which they can be enzymatically digested, generally by trypsin, through in‐gel digestion or in‐solution digestion. For in‐gel digestion, trypsin is allowed to penetrate into the dried gel pellets and digest the protein in the gel. After this, the tryptic peptides, much smaller than the original proteins in the gel, can easily be extracted from the gel, vacuum‐dried, and tested by MS. When loaded onto a MALDI plate, the samples will be covered with a matrix such as CHCA, for downstream MS analysis 18. Bacterial extract for routine MALDI‐TOF‐MS test can also be digested, fractionated through chromatography, and loaded on MALDI plate to reduce the sample complexity on each MALDI spot, and further fragmentation of the selected masses can be performed to get peptide sequences and subspecies level identification 19.

Recently, MALDI‐TOF mass fingerprinting was also used to detect fatty acids on bacteria 20. Gram positive or negative bacteria from pure culture were suspended in phosphate buffer saline and then mixed with chloroform/methanol to extract the fatty acids out for MS detection. Positive‐ and negative‐ion modes were used and the fatty acids were shown closely related to species level identification of the bacteria.

Mycolic acids (MAs) detection in *Mycobacteria* infection, especially from tuberculosis (TB), has been tried recently by LC‐MS/MS profiling of MAs. Sputum samples were diluted in basic buffer and then treated with chloroform/methanol to extract MAs. The method showed high sensitivity and specificity for TB diagnosis 21, 22.

MALDI‐TOF‐MS has also been recently reported to be able to detect carbapenemase or β‐lactamase activities, an indicator of antibiotics hydrolysis, by incubating the bacteria from positive blood culture with the enzyme substrate (antibiotics). If the substrate gets hydrolyzed showing mass shift in MS detection, the antibiotics resistance can be suspected 23, 24. LC‐MS method has also been used for this antibiotics susceptibility test to check the antibiotics and its metabolite profiles. For example, ampicillin (*m/z* 350 Da) can be hydrolyzed into ampicillin–penicilloic acid (*m/z* 368 Da) and penilloic acid (*m/z* 324 Da) in ampicillin‐resistant cells, and these mass changes can be easily detected by LC‐MS 25.

### 2.3 Peptide sequencing for protein identification by LC‐MS/MS

**Figure 3:**
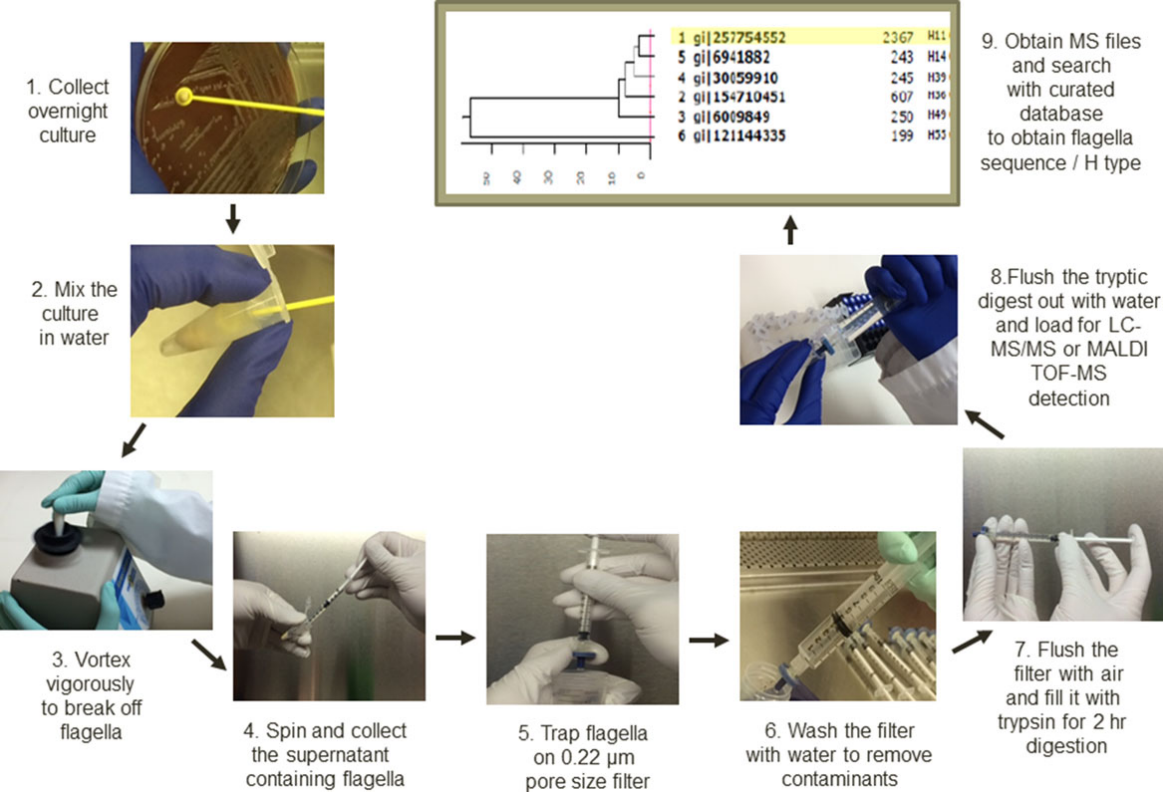
MS‐based flagella typing (MS‐H) workflow.

Peptide amino acid sequences are more often obtained with LC‐MS/MS systems. Whole proteome or proteins of interest are digested, separated by HPLC, and the ionized peptides are detected and further fragmented. Peptide sequences will be obtained by comparing the tandem spectra to the theoretical peptide fragmentation spectra. For example, Kooken et al. reported that bacterial proteins could be extracted by physically breaking the cells and separated by SDS‐PAGE. In‐gel digestion would be performed and LC‐MS/MS would then be used to check the bacterial protein bands of interest 26. The authors claimed that even with sequence information obtained by LC‐MS/MS, correct database was still important for correct identification. Paul et al. reported that enriched *Clostridium botulinum* flagella were pelleted, washed, centrifuged, and run on SDS‐PAGE for trypsin digestion and peptide sequencing 27. A method has been developed by our group to enrich *E. coli* flagella by shearing flagella out of the cell body and trap them on a filter membrane. On‐membrane digestion is then performed and MS detection of the digest will then be carried out for sequence‐level identification and typing of all *E. coli* flagella [28, Fig. 3]. Enrichment of molecules of interest is very important to have high sequence coverage 27, 28. For general LC‐MS/MS testing of whole bacterial proteins, abundant proteins should provide almost a complete coverage and give more confident identification while less abundant proteins will be buried in the chromatograms 26, 29. The advantage of using LC‐MS/MS is that detailed information and minor differences among the isolates at subspecies level, or at different growth conditions for the same isolate, can be observed 26, 27, 28, 29, 30.

### 2.4 Targeted LC‐MS/MS for protein identification and quantitation

Targeted protein identification and quantification can be performed by MRM through a quadrupole MS system 31. Ions (charged peptides) of interest can be selected from one quadrupole field and fragmented in the next quadrupole field. The sequences of the peptides will then be obtained, and the fragmented ions can be used for quantitation if stable isotope‐labeled standard peptides are used as identification and quantitation references. In MRM, ion selection and fragmentation (MRM transition) is very fast and hundreds of targeted proteins can be identified and quantified in a 1‐h run of LC‐MS/MS. Either protein standards or peptide standards can be synthesized and used 32, 33. The advantage of using protein standards is that they can be spiked into the testing sample to be processed, and the quantification of the protein can be performed accurately since the standards go through the sample preparation process 32. MRM is highly specific, sensitive, accurate and reproducible. Rees et al. used the unique affinity of organisms for their host, such as bacteriophages specific for certain bacteria, and checked the phage amplification product through sequence‐based detection methodologies of LC‐MS/MRM to deduce the antibiotic resistance properties of the host cells since the phage could only amplify if the host cells could grow in the presence of the antibiotics 34. MRM‐based quantitation can be multiplexed to analyze and quantitate many molecules of interest per sample run, increasing the throughput of the assay, which makes it more desirable for clinical applications 33. A recent publication by Charretier et al. showed that with *Staphylococcus aureus* as a model, bacteria identification, antibiotics resistance, virulence, and type profiling could be all obtained using one MRM platform 35.

In addition, targeted LC‐MS/MS can also be applied for MA detection and structural confirmation. Selected masses of MA were further fragmented, and MA‐specific ions (masses) will be detected 21, 22. This is different from sequence‐based peptide identification and quantitation by MRM, but for specific structure confirmation on MAs, a group of fatty acid‐like molecules that are unique to *Mycobacteria*. This MRM‐based test on MAs was much faster than traditional diagnosis for TB, for which the slow growth of *Mycobacteria* is a significant problem for rapid diagnosis 21, 22.

There are very few limits on the variety of samples that MS can analyze. As seen in the following sections, there are many studies on various types of bacteria that are of importance to human health. They all have unique surface molecules, marker proteins, or toxins that allow them to be differentiated, identified and analyzed by MS.

## 3 MS and proteomics applications on common pathogenic bacteria

### 3.1 Escherichia coli (E. coli)

*Escherichia coli* are well known for human enteric diseases, urinary tract infections, bacteremia, and intestinal infections even though most strains are nonpathogenic 36, 37. *Escherichia coli* serotyping, the current gold standard, is time consuming due to the induction of flagella growth and multiple steps required 18. PCR‐based detection and multilocus sequencing typing (MLST) on several housekeeping genes 18, 36 are not phenotypic typing methods to differentiate motile strains (with flagella) and nonmotile strains (without flagella). Results indicate that many pathogenic types of *E. coli* are not routinely observed due to a lack of rapid and thorough methods 37. Current studies show that MS is a rapid and simple detection method that provides accurate results 18, 37, 38. Clark et al. utilized the MALDI‐TOF‐MS and the identified protein peaks were unique at the genus and species level, and MALDI‐TOF‐MS mass spectra were quite reproducible. The study searched for specific diagnostic peaks rather than using the total spectrum for isolates differentiation, and the MS peaks chosen for *E. coli* identification were stable and sufficiently robust 37.

An LC‐MS/MS‐based method, named MS‐based H typing (MS‐H), came out recently from our lab for H antigen (flagella) typing of *E. coli* through on‐filter flagella enrichment and trypsin digestion, and LC‐MS/MS‐based peptide sequencing [Fig. 3]. The obtained flagellin peptide sequences were used to identify *E. coli* flagella with a curated *E. coli* flagellin database. Clinical scenario was simulated, and in one report, a total of 127 clinical isolates were preliminarily tested and 90.5% of the results were in agreement between traditional serotyping under motility induction and MS‐H typing without motility induction, and MS‐H could type some isolates that were “undermined” in serotyping 38. Recently, we also identified all *E. coli* flagella types using PMF on a MALDI‐TOF‐MS system 18. MALDI‐TOF‐MS platform was certainly faster with higher throughput than LC‐MS/MS platform, but due to the limited sample loading amount on individual MALDI plate spot, the PMF result from MALDI‐TOF‐MS was not as consistent as LC‐MS/MS platform, which could enrich peptides by HPLC and gave flagella types at sequence level. Repeated MALDI‐TOF‐MS tests with multiple spots per test were recommended to obtain reliable results.

Fagerquist et al. used top‐down method to identify *E. coli* Shiga toxin 2 by MALDI‐TOF‐TOF tandem MS for intact toxin‐specific peaks without protease treatment of the cell lysate after overnight induction of the toxin expression with chemicals such as ciprofloxacin or mitomycin C. In‐house developed software was used for the toxin identification, and the result of 26 isolates tested matched DNA sequences of the toxin well 39.

### 3.2 Salmonella

*Salmonella* are common food‐borne pathogens, which may be lethal if consumed. Detection and identification is highly necessary in food sources and for surveillance of salmonellosis 40, 41. There are more than 2500 serovars of *Salmonella*, which are traditionally identified by serotyping, also a time‐consuming process. For dual phasic H antigens, a process called “phase suppression” has to be used to delineate the two phases 41. MALDI‐TOF‐MS/MS has been used recently based on its rapid speed, ease of operation, and cost effectiveness, and it could reach genus and species level identification for *Salmonella* 19. Top‐down approach can also be used for intact mass measurement and differentiation among *Salmonella* subspecies by LC‐MS‐based mass profiling 40.

We also explored MS‐H typing on *Salmonella* flagella [41, Fig. 3]. Curated *Salmonella* flagellin database was used to identify flagella types. The method was compared to traditional serotyping and was shown to be faster and easier. Twenty‐four serovars from 43 strains of the most common types of *Salmonella* were selected. The tests contained 17 monophasic isolates, 25 diphasic, and one triphasic. This novel method accurately identified 100% of all monophasic *Salmonella* strains at the individual H antigen level, 100% of diphasic strains at the antigen cluster/complex level, and among the diphasic strains 75% accuracy for phase 1 antigens and 69% accuracy for phase 2 antigens.

## 3.3 Campylobacter jejuni

*Campylobacter jejuni* is the leading cause of bacterial gastroenteritis worldwide and can cause Guillain‐Barré syndrome 6, a severe neurological syndrome 42. There are several methods for identifying/typing *Campylobacter*, antibody‐based slide agglutination on heat stable and heat liable antigens, MLST and recently, MALDI‐TOF‐MS for intact cell MS detection 6. Zautner et al. determined whether MALDI‐TOF‐MS could differentiate between subtypes of *C. jejuni*. They utilized 104 previously characterized *C. jejuni* isolates from humans, chicken, turkey, and bovine. All samples were identified as *C. jejuni* by MALDI‐TOF‐MS Biotyper, and the method discerned mass peaks to identify subtypes, for example, *C. jejuni* subspecies *jejuni* and *C. jejuni* subspecies *doylei*. The statistical method called phyloproteomic principal component analysis hierarchical clustering was used to categorize MALDI‐TOF‐MS data and showed it closely reflected phenotypic aspects 6.

### 3.4 Clostridium

*Clostridium* includes species that produce toxins, are clinically important, and useful for industry 43. *Clostridium botulinum* produces the highly toxic botulinum toxin 44 and four serotypes of the toxin, A, B, E, and F are known to cause disease in humans 45. *Clostridium difficile* produces at least three toxins, causes infections in humans and animals, and is the leading cause of antibiotics‐associated diarrhea worldwide 46. Current detection methods include biochemical tests, GC, PCR 43, and immunoassays on stool sample 46. Animal methods have also been employed to detect toxins, for example, the mouse bioassay 45. However, these methods are time consuming and do not consistently provide easy and accurate identification 43. MS‐based analysis is gaining popularity since prior knowledge of the bacteria is not necessary before analysis, a brief sample preparation is all that is required, and it is more cost effective than having to maintain animals 43, 44, 45, 46. Identification and differentiation of *Clostridium* species were analyzed through MALDI‐TOF‐MS fingerprinting by Grosse‐Herrenthey et al. and the study showed sample preparation was simple and only a single colony of cell culture was required for the analysis and MS results were obtained within minutes. They utilized 64 strains with 31 species and found unique mass fingerprints for all samples including *C. novyi* types A and C and *C. botulinum* types C and D, which were not differentiated through biochemical testing 43. Kalb et al. and Wang et al. used an Endopep‐MS method, a MALDI‐TOF‐MS‐based method, to analyze the botulinum toxin 44, 45. Botulinum toxins are endopeptidases and are highly substrate specific. The Endopep‐MS method is based on the masses of the cleavage products, as they will be unique based on which toxin is present. The authors focused on the light chain of the toxin, providing it with a substrate in vitro that mimics the in vivo substrate. The presence/absence of the toxins was detected with 100% accuracy, and the analysis was not disturbed by the complex sample matrices analyzed such as meat and milk. The method also worked if multiple toxins were present in one sample based on the unique cleavage products. A stringent wash step with 2 M NaCl was applied after the substrate was added, and the toxin was allowed to adhere to the antibody‐coated beads. This wash helped reducing nonspecific binding. In addition, alterations on the toxin substrate such as single and multiple amino acid substitutions, incorporation of unnatural amino acids, terminal modifications and length determinations were performed, and the authors concluded the novel peptide substrate allowed for increased detection of botulinum toxins. All toxins were detected within 4 h in complex matrices such as serum and milk. The LOD was determined in each matrix, buffer, serum and milk, and they were 0.5, 0.5, and 1 mouse lethal dose_50_ (LD_50_), respectively 45.

Moura et al. utilized two methods of LC‐MS/MS to detect the presence of *C. difficile* toxins in cell culture filtrate, ultraperformance LC‐MS/MS (UPLC‐MS/MS) and data‐independent UPLC‐MS/MS 46. The difference was that the data‐independent method was a label‐free method that could determine the protein identification and quantification in one MS experiment using alternating high and low energy. The UPLC‐MS/MS confirmed that digestion with trypsin was efficient and robust with enough amino acid coverage for identification of the two main *C. difficile* toxins, TcdA and TcdB. They also showed that the most efficient combination of enzymes for differentiation of the two toxins was trypsin and GluC, providing amino acid coverage of 91% for TcdA and 95% for TcdB. The data‐independent method quantified toxins at low levels and identified them separately. This is a novel development as conventional methods typically quantify the total amount of toxin present. The method detected TcdA at 5 ng (1.6 μg/mL) and TcdB at 1.25 ng (0.43 μg/mL). Cell culture filtrate appears to be a novel approach for detection of *C. difficile* toxins creating potential for future study.

### 3.5 Listeria monocytogenes

*Listeria monocytogenes* is an opportunistic pathogen found in vegetables, meat, dairy, and poultry, and causes approximately 2500 infections per year in the United States. It is the major cause of listerosis primarily affecting immunocompromised patients and pregnant women. *Listeria* are serotyped based on O and H antigens and current identification methods include PFGE, MLST, and PCR, however these methods can become laborious. Barbuddhe et al. used MALDI‐TOF‐MS to analyze *Listeria* samples to determine whether mass fingerprinting could subtype the isolates 47. A short and simple protein extraction before MALDI‐TOF‐MS analysis was performed and results indicated all 146 isolates were correctly identified at the species level, and MALDI‐TOF‐MS analysis was confirmed through 16s RNA sequencing identification. The reproducibility was also tested as samples were analyzed at over several hours, on different MALDI‐TOF‐MS instruments, with different batches of culture media, after multiple days of incubation, and after storage at −20°C for weeks. All analyses provided similar results, indicating the reproducibility was quite substantial. The authors did claim that sample preparation to extract proteins from *Listeria* was critical before analysis on MALDI‐TOF‐MS.

### 3.6 Mycobacteria

*Mycobacterium tuberculosis* is a global health concern with approximately 2 million deaths each year 21. It is increasingly problematic as strains become multidrug resistant 48 and many non‐TB *Mycobacteria* are emerging as serious pathogens. They are able to survive on and within medical equipment and infections are increasing, especially among immunocompromised patients 49. Currently, TB is diagnosed through acid‐fast bacillus (AFB) staining and MAs are the target for detection as they are unique to *Mycobacteria* 21.

LC‐MS/MS has been used to detect MAs. Shui et al. applied this method, involving MRM analysis, directly on sputum samples. Minimal sample preparation was required and same day results were obtained, providing faster diagnosis/treatment for patients compared to traditional methods, as culturing, which could take 6–8 wk, was not required. The sensitivity was 94% and the specificity was 93%, compared to 60% sensitivity and 95% specificity in AFB for which other organisms could lead to positive results since it relies on microscopy, which provides opportunity for human error 21. Szewczyk et al. also detected MAs through targeted LC‐MS/MRM directly from sputum. Ten MRM transitions were used, and the electrospray needle was washed with chloroform for 3 s after each sample run to avoid carryover. If same day detection was not possible, the isolates would be cultured for ten more days. Sixteen samples from TB patients were analyzed and MAs were detected in 11 of 16 samples without cell culture and 15 of 16 samples after the 10‐day culture. MAs were also detected by MS in samples determined negative by AFB. The sensitivity was 69% for direct detection and 94% after the culture. Further tests were performed with *Corynebacteria* to determine if the analysis was specific for *Mycobacteria* and no false‐positive results were reported 22.

Wilen et al. interchanged three different MALDI‐TOF‐MS instruments and sample preparations to observe the instrument's ability to identify TB and non‐TB *Mycobacteria*: Biotyper, VITEK MS, and Saramis 49. The majority of the 157 isolates were correctly identified by all systems; Biotyper identified 84.7%, VITEK MS 89.2%, and Saramis 85.4%. When sample preparations were interchanged, misidentifications did not increase but lower confidence scores for proposed identities were observed. Although the instruments could tolerate alternate sample preparations it was best to prepare samples in the same manner as instructed, as the instruments databases contain information from samples prepared and analyzed in the particular method provided. Dunne et al. tested *Mycobacteria* that were inactivated and subjected to a variety of conditions such as freezing and thawing, refrigeration, and increased time of incubation on culture media to determine whether MS results would be affected. MS peaks were similar in all cases, suggesting MALDI‐TOF‐MS could handle samples from a variety of conditions without compromising the integrity of the results, which was beneficial as this indicated samples could be frozen and shipped or stored over a period of time and the analysis would still be reliable 50.

Leprosy, the chronic disease caused by *Mycobacterium leprae*, is still a severe public health concern and remains endemic in tropical and undeveloped regions of the world. It affects epidermal cells and peripheral nerves, which results in severe skin lesions, disabilities, and social stigma 51. Multiple conditions must be met before a definitive diagnosis is made, and current guidelines are not very clear, especially in suspected cases. Conventional diagnostic procedure depends on skin smear microscopy and histopathology of skin biopsy, which is invasive, uncomfortable, and results in slow diagnosis with low sensitivity. A new method developed by Lima et al. utilized silica plates gently pressed and held against a skin lesion. The lipid composition of infected skin was supposed to be different than of healthy skin, phospholipid and sphingolipid composition specifically. The silica plate‐absorbed lipids from the skin were then extracted and collected using methanol. Samples were analyzed by ESI high‐resolution MS (ESI‐HRMS). MS identified signals that could differentiate healthy skin from the skin lesions. It also identified differences between adjacent unaffected skin close to the lesion and healthy skin. This noninvasive method allowed for earlier diagnosis in cases that are not yet presenting typical symptoms, resulting in earlier treatment 51.

### 3.7 Other bacteria or combinations of bacteria in clinical matrices

*Staphylococci* are Gram‐positive bacteria that cause pus‐forming infections, food poisoning, and are the major cause of wound infections, nosocomial acquired pneumonia, and septicemia 29, 52, 53, 54. Currently, different results were obtained for *Staphylococci* species identification by MS approaches 26, 29, 52, 53. Even so, evidence showed that the emergence of multiple *staphylococcus* strains resistant to prescribed antibiotics could be detected by targeted LC‐MS/MS by incubating bacteriophage with the bacteria 34. In addition, Hennekinne et al. used isotope‐labeled protein standards spiked into food extracts to identify and quantify enterotoxins in *S. aureus* food‐poisoning outbreak with LC‐MS/MS platform. Bacterial toxins were enriched by immune‐affinity method and run on SDS‐PAGE. In‐gel digestion was performed and the toxin peptides were identified and quantified based on the quantitation standards 54.

*Helicobacter pylori* is a Gram‐negative bacterium, the primary cause of active chronic gastritis, peptic ulcer disease, and an important risk factor of gastric cancer in humans as it is the only bacterium that can colonize the human stomach 55, 56. In an experiment by Xiao et al., the MALDI‐TOF‐MS Biotyper system was shown to identify *H. pylori* well, and a new method based on PMF was developed and used for identification of two *H. pylori* types, P1 and P2. Specific peaks were determined to differentiate the two types 55. A study by Zhou et al. provided the first profile characterization of lipid A component from a single colony of *H. pylori* through MS. The new method utilized a microwave‐assisted enzymatic digestion and detergent‐free mild hydrolysis in conjugation with an MALDI‐TOF‐MS analysis, and strain‐specific characteristics allowing for differentiation and detection of mutant isolates were found 56.

Richter et al. tested the ability of the VITEK MS to identify enterobacteriaceae typically encountered within the clinical laboratory. Seventeen genera including 40 species totaled 965 isolates were directly smeared on MALDI plate for identification 15. The VITEK MS system identified 83.8% of isolates to the species level, 12.8% to the genus level, but 0.7% was misidentified, and 1.7% produced no identification. Although the method utilized direct loading of samples onto the MALDI plate, required fewer reagents and produced faster results, there appeared to be a bias in the efficiency of identification. Some species would be identified accurately and consistently and some species would not be able to be identified at all. This was likely due to the presence or absence of species in the database.

He et al. tested the ability of Biotyper on enteric pathogens from colonies grown on selective stool culture media 7. Colonies were directly smeared onto the MALDI plate, and if identification was not successful a protein extraction step was performed to improve results. The experiment included 304 isolates, and phenotypic methods determined that 68 strains were pathogenic bacteria, and 236 were normal flora. The Biotyper analysis correctly identified 22 *Salmonella* species, two *Yersinia enterocolitica* isolates, and two *Campylobacter* species; however it misidentified 39 *Shigella* species as *E. coli*. This result supported the claim in the previous study that MALDI‐TOF‐MS could not differentiate between *Shigella* and *E. coli* 15. Even so, the authors claimed MALDI‐TOF‐MS could reduce labor and turnaround time by 2–3 days. The cost was also reported to be reduced since traditional stool‐based culture detection methods were costly and require multiple days, while MALDI‐TOF‐MS cost much less 7, 57.

Van Veen et al. went into great detail with a wide variety of isolates tested by MALDI‐TOF‐MS. Enterobacteriaceae, nonfermentative Gram‐negative rods, Gram‐positive cocci, yeast, and “other Bacteria,” were analyzed through retrospective and prospective studies 58. The retrospective study involved 327 isolates directly smeared onto the MALDI plate, and if direct colony smear did not produce confident results, a simple sample preparation was utilized. The first observation was that yeast cells always required the sample preparation if a correct identification was to occur. This step also helped the identification for nonfermentative Gram‐negative rods. Interestingly, it was not required for Gram‐positive bacteria or enterobacteriaceae. Of these 327 isolates, 280 were identified to the species level, 24 were identified to the genus level, but ten isolates were misidentified, seven isolates did not produce uniform identities across repeated tests, and six isolates produced no results at all. Some of the misidentifications were due to species being absent from the database. In the prospective study, MALDI‐TOF‐MS was able to make more species identifications than the conventional methods (92% for MALDI‐TOF‐MS vs. 83.1% for conventional method). The authors concluded that MALDI‐TOF‐MS analysis performed identification as good or better as conventional methods, and MALDI‐TOF‐MS had the benefit of providing faster results as well as providing a higher throughput, but there appeared to be a trend indicating a bias though: MALDI‐TOF‐MS had a much higher percentage of correct identification for enteric bacteria and struggled more to identify Gram‐positive cocci in particular. This was likely due to the major limitation of the MALDI instrument, it was only as strong/weak as its database. The authors did say that MALDI‐TOF‐MS was able to identify coagulase negative *Streptococci* isolates to the species level better than other methods and that they had implemented this mode of identification into their routine work 58.

MALDI‐TOF‐MS platform has been recently tried on some rare bacteria such as *Avibacterium, Myroides, Granulicatella*, and *Abiotrophia*, with comparable results and advantages 59, 60, 61. In addition, recent reports have shown that both identification and antibiotics susceptibility test results can be obtained together in a few hours from positive blood culture by MALDI‐TOF‐MS 23, 62, 63, 64. Urine microbial identification could also be performed with minimum cell culture after centrifugation and filtration steps to collect the bacteria 64, 65, 66. Cerebrospinal fluid was also used to diagnose meningitis after cell culture 67, 68.

## 4 Reflection of pro and cons of MS platforms and their use on solving hard problems in bacteriology

One of the widely noted benefits of MALDI‐TOF‐MS applications mentioned in nearly every related article cited here was that this platform produced incredibly fast results, in some cases just a few minutes after samples were loaded on the systems. MALDI‐TOF‐MS regularly utilized fewer reagents, fewer steps and required less prior information about the organism than methods such as PCR or biochemical tests. As a result of the faster data acquisition time, the overall analysis was also cheaper since it occupies fewer working hours than the traditional methods and the cost per MALDI‐TOF‐MS sample was reported to be approximately $0.50–1.00 69. The speed of obtaining results from MS would be a great benefit for epidemiological studies and clinical diagnosis where time is of the essence and faster identification will ultimately benefit patients 70.

MALDI‐TOF‐MS was also noted to be quite reproducible and reliable by some users 70. The use of MALDI‐TOF‐MS for identification also reduced the chances of human error that can result from interpretation or judgment calls to determine the results when identification relied on phenotypic, biochemical, or microscopic analysis.

As with all new methods there are some less than desirable effects that still have to be worked out in MALDI‐TOF‐MS‐based platforms. The first is the high initial cost that comes with purchasing the instrument. The other major limitation of MALDI‐TOF‐MS is the requirement of a database. The results will only be as accurate as the database available, which becomes problematic when dealing with obscure species or emerging pathogens where data might not be as available as more commonly studied species 59. Following this, there are also studies showing MALDI‐TOF‐MS had difficulties differentiating closely related species, with reports that the identification was only confident to the genus level 58. Some studies also reported that the culture media, time, and temperature also affected how well MALDI‐TOF‐MS could identify strains 13, 71. This topic leads to some controversial results as some studies report excellent differentiation up to the subspecies level and some studies report that the MALDI‐TOF‐MS was unable to identify particular isolates at all. One report suggested that culture method be optimized for each organism that was to be identified by MALDI‐TOF‐MS, but this has to be done on an individual basis as there is not one general cultivation media, temperature, or time that would be optimal for all isolates 71. We agree with recommendations by experts that more initiatives should be taken to refine the databases, especially on some rare species of bacteria, through the collaborations among public health laboratories who may have a complete list of bacteria at subspecies level, and instrument venders who finally provide the databases for customers 72, 73. In addition, more efforts should be put to harmonize the protocols for sample preparation among laboratories that routinely perform MS‐based bacteria identification and typing. With more popularity of whole genome sequencing on microorganisms, more understanding of the genomic similarities/differences can be obtained. This will help explain some discordant result in MALDI‐TOF‐MS‐based bacteria identification. For example, *E. coli* and *Shigella* are very similar in their genome compositions 74, which might indicate why we could not easily differentiate them in MALDI‐TOF‐MS detection.

There are many more reports on the MS‐based applications for antibiotics susceptibility/resistance tests either by MALD‐TOF‐MS 16, 23, 24, 75, LC‐MS 25, or targeted LC‐MS/MS 34, 35. This is another trend that MS will revolutionize microbiological testing in the future.

## 5 Conclusion and prospective

An appealing aspect of MS is that it is not limited to what it can detect or the applications that it can have. Any molecule that can be ionized has the potential to be detected by MS with high sensitivity and resolving power. Current studies have confirmed that MS can analyze any cultivable organism and its related metabolites without much prior knowledge, and it has shown useful in a variety of applications, from fast hospital diagnosis and identification of traditional bacteria, to organisms that are difficult to culture. With the gaining popularity of MS instrumentation, growing detectability, and more user friendly MS hardware and software, MS will certainly play more positive roles in solving critical problems such as antibiotics resistance and slow‐growing bacteria that are difficult to identify quickly by traditional approaches.

5.1

*This project was supported by Genomics Research and Development Initiative (GRDI) funding from the Public Health Agency of Canada*.

5.2

*The authors have declared no conflict of interest*.
